# Investigation of the Relationship between the Increase in the Intercanine width and the Children’s Facial Parameters; a 6-month Follow-up Study

**Published:** 2013-06

**Authors:** F Ghaderi, S Badakhsh, S Hekmatfar

**Affiliations:** a Dept. of Pediatric Dentistry, School of Dentistry, Shiraz University of Medical Sciences, Shiraz, Iran.; b Dept. of Pediatric Dentistry and Committee Dental Research, School of Dentistry, Zanjan University of Medical Sciences, Zanjan, Iran.; c Dept. of Pediatric Dentistry and Committee Dental Research School of Dentistry, Ardabil University of Medical Sciences, Ardabil, Iran.

**Keywords:** Intercanine Width, Facial Parameters, Increase

## Abstract

The present study, as a pilot study, aimed to investigate the increase in the intercanine width in different facial forms to predict the amount of future increase in the intercanine width. The results of the pilot study showed that the intercanine width increased more in the boys with wider faces while this relationship was not observed in the girls. Based on the results of this preliminary study, the girls’ facial width could not be considered as a determining criterion in evaluation of the amount of increase in the intercanine width.

## Introduction

Anterior dental crowding, in mixed dentition, is a common challenge for dental practitioners. Today, parents are concerned about this problem and the possibility of its elimination and the need for future orthodontic treatment is among their primary enquiries [1]. As the permanent mandibular lateral incisors erupt, a 2-3 mm increase is observed in the intercanine width. In some cases, however, due to the inadequate intercanine growth, stripping and even extracting the primary canines is required to limit the crowding [2]. Therefore, the prediction of the changes (in terms of quantity) in the dental arch as well as the intercanine width is important in children. This is based on the facial parameters in the first visit to determine the kind of requisite measurements and the ideal time for the treatment of dental crowding in the early mixed dentition. 

Various studies have been conducted on the intercanine width and its relationship with the arch length, facial height and so on [3-5]. They showed long-face individuals to have narrower transverse dimensions in comparison with the short-face ones. To the best of authors’ knowledge, few studies have been conducted on the amount of increase in the intercanine width in different individuals. Moorress et al [5] reported a higher increase in the maxillary intercanine width in the boys compared to the girls and lower probability of crowding in the boys’ mandible. Since the amount of increase in the intercanine width is imperative in the elimination of the anterior crowding, the prediction of the intercanine width is important in different individuals [2].

Until now, no studies have been conducted about the predication of the amount of the increase in the intercanine width based on facial forms. So, the present study, as a pilot study, aimed to investigate the increase in the intercanine width in different facial forms to predict the amount of future increase in the intercanine width.

## Material and Methods

This pilot study was conducted by recruiting 32 children with the age range of 7-8 years old (the age before the lateral incisor eruption) who had been selected from the primary schools of Shiraz, Iran with the aid of the cluster sampling.

**Figure 1a:**
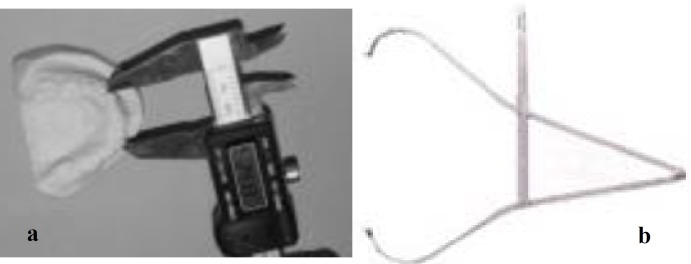
Measuring the intercanine width **b **ICS-Spreading Caliper-bow

After measuring the facial dimensions by caliper bow (ICS-Spreading Caliper-SPCG01P, Swiss) (figure 1a), mandibular mold was made by alginate (Tropicalgin; Zhermack clinical, Italy) and the dental cast was immediately prepared by using the stone plaster (Hinridurs; Germany). The intercanine width was measured and recorded from the cusp tip of the canine of one side to the cusp tip of the other side canine through a digital caliper (Shoka gulf; Spain) (figure 1b). Facial dimensions were measured by the caliper bow as follows: 

1. Measurement of facial height (from Nasion to Menton in soft tissue)

2. Facial width (the distance between the most prominent points of the zygomatic bones in the frontal view).

Moreover, to determine the amount of the increase in the intercanine width, the children were visited six month later and the intercanine width was measured and recorded. 

Collected data were analyzed statistically by adopting the SPSS version15. Student t-Test and the correlation coefficient test were also performed.

## Result and Discussion

A significant correlation was observed between the increase in the intercanine width and the facial width in the boys (r= 0.624, *p*= 0.031); however, this relationship was not observed in the girls.

As mentioned before, no studies have investigated the increase in the intercanine width and its relationship with facial parameters until now. In the current pilot study, the intercanine width increased more in the boys with wider faces while this relationship was not observed in the girls. This might be because of the masseter and temporalis muscles, which in the girls tend to be thinner than the boys [6-7]. So, based on the results of the present study, the girls’ facial width could not be co-nsidered as a determining criterion in evaluation of the amount of increase in the intercanine width (Table 1). 

**Table 1:** Average increase in the intercanine width in both sexes during the 6-month follow-up period

**Sex**	**Amount of increase in the intercanine width (mm)**
Girl	0.94
Boy	0.79

Further studies with longer follow-up periods and larger sample sizes are needed to be conducted on the subject of this pilot study.
